# Chronic respiratory symptoms and associated factors among cement factory workers in Dejen town, Amhara regional state, Ethiopia, 2015

**DOI:** 10.1186/s40248-016-0043-6

**Published:** 2016-03-01

**Authors:** Zemichael Gizaw, Bamlaku Yifred, Takele Tadesse

**Affiliations:** Department of Environmental and Occupational Health and Safety, Institute of Public Health, College of Medicine and Health Sciences, University of Gondar, Gondar, Ethiopia; Labor and Social Affairs Office, East Gojam Zone, Amhara National Regional State Northwest Ethiopia

**Keywords:** Cement dust, Chronic respiratory symptoms, Occupational exposures

## Abstract

**Background:**

Chronic respiratory diseases represent a public health challenge in both industrialized and developing countries. Chronic respiratory symptoms are more prevalent in cement factories of developing countries, where occupational health and safety issues are less emphasized.

This study was conducted to determine the prevalence and factors affecting chronic respiratory symptoms among workers in Dejen cement factory, 2015.

**Methods:**

Institution based cross sectional study was conducted among 404 randomly selected study participants. Data were collected through interviewer administered structured questions derived from British Medical Research Council (BMRC) adult respiratory symptom assessment questions and observational check lists for the assessment of dust exposure, hygienic practices and use of personal protective equipments. Multivariable logistic regression model was used to identify predictor variables which have association with chronic respiratory symptoms and finally the variables which had significant association were identified on the basis of Adjusted Odds Ratio (AOR) with 95 % Confidence Interval (CI) and p < 0.05

**Results:**

The prevalence of chronic respiratory symptoms among Dejen cement factory workers was 62.9 %, with prevalence of chronic cough 24.5 %, chronic wheezing 36.9 %, chronic phlegm 24.5 %, chronic shortness of breath 38.6 %, and chest pain 21.0 %. Chronic respiratory symptoms were associated with sex (AOR = 2.07, 95 % CI = 1.18, 3.63), age (AOR = 4.20, 95 % CI = 1.94, 9.12), education level (AOR = 4.07,95 % CI = 1.86, 8.92), cement mill (AOR = 3.72, 95 % CI = 1.92, 7.21), burner and clinker (AOR = 2.28, 95 % CI = 1.18, 4.43), work experience (AOR = 5.44, 95 % CI = 3.09, 9.59), training on occupational safety and health (AOR = 2.73, 95 % CI = 1.41, 5.29), smoking (AOR = 5.38, 95 % CI = 1.42, 20.39) and chronic respiratory diseases (AOR = 7.79, 95 % CI = 2.02, 30.04).

**Conclusion:**

Chronic respiratory symptoms were highly prevalent among Dejen cement factory workers. Age, sex, education level, working department, smoking, work experience, and training were identified factors. Pre employment and on service training, smoking cessation programs, improving hygienic practices are important tasks in order to maintain the health and safety of workers.

## Background

Chronic respiratory symptoms such as chronic cough, chronic phlegm, wheezing, shortness of breath, and chest pain are manifestations of respiratory problems that are mainly developed as the result of occupational exposures [1]. Chronic respiratory diseases represent a public health challenge in both industrialized and developing countries because of their health and economic impacts [2].

In 2012 World Health Organization (WHO) reported that worldwide non-communicable diseases are the leading cause of mortality which accounts for 82 % of deaths and among those non-communicable diseases chronic respiratory diseases, asthma and chronic obstructive pulmonary diseases accounted for 4 million or 10.7 % deaths [3].

Occupational respiratory diseases account for up to 30 % of all registered work related diseases and 10 - 20 % of deaths are caused by respiratory problems. Occupational respiratory diseases account for up to 50 % prevalence among workers in high risk sectors such as mining, construction, and dust generating works [4].

In developed nations like Great Britain there are currently about 12,000 deaths each year due to occupational respiratory diseases, about 2/3 of which were due to dust related diseases [5].

In developing countries like India, the second cement producing country, respiratory disease accounted for 17% of the 11 million occupational diseases and chronic obstructive pulmonary diseases are responsible for 87 % of work related respiratory disease mortality [6].

In low income countries, especially sub- Sarah African countries, respiratory problems are the sixth cause of death and most of these problems are due to dust exposure associated with increasing cement factories [7, 8].

Dust is one of the main occupational hazards that causes chronic respiratory problems in cement manufacturing industries. Workers in cement factories are exposed to different health hazards during cement production and handling, including cement dust, high temperature, and noise. However, the major occupational hazards in the cement production industry are cement particles which are emitted to the environment at most stages of production process with higher concentration in the packing and crusher section [9–11].

Studies in fast developing Asian countries reported that prevalence of respiratory symptoms are increasing and associated with cement dust exposure [12–14].

## Purpose of the study

This study was conducted to assess prevalence and associated factors of chronic respiratory symptoms among cement factory workers in Dejen town, 2015.

## Methods

### Study design

An institution based cross sectional study was conducted to assess prevalence of chronic respiratory symptoms and associated factors among workers in Dejen cement factory.

### Source and study population

All workers (night and day shift) recruited in Dejen cement factory were the source population but all workers who were directly involved in the production and cleaning activities and who had worked more than one year were taken as study population, whereas workers who had less than one year work experience in the same job were not included in this study.

### Sample size determination

The sample size was determined by using single population proportion formula with the following assumptions:

- p: The proportion in the population with chronic respiratory symptoms = 66.20 %
- w: (Margin of error or level of precision or maximum error to commit) = 5 %
- 95 % Confidence interval (standard normal probability)
- z: The standard normal tabulated value
- α: Level of significance

$$ n=\frac{{\left({z}_{\frac{\alpha }{2}}\right)}^2p\left(1-p\right)}{w^2}=\frac{(1.96)^20.662\left(1-0.662\right)}{0.05^2}=345 $$

For internal comparison, sample size was calculated for the most important determinant factors (Table 1). The sample size calculated for training on respiratory symptoms was larger than the sample sizes calculated for other associated factors. Therefore, the sample size was taken as 384. By considering 5 % non response rate, the final sample was 404.

**Table 1:** Sample size determination for associated factors for chronic respiratory symptoms

No-	Associated factors	Proportion of population with respiratory symptoms (p)	Odds ratio	Sample size obtained
1	Utilization of personal protective equipment (PPE)	55 %	2.04	380
2	Training on respiratory health and safety	51 %	0.18	384
3	Educational level	31 %	0.15	328

### Sampling procedures

Hence, the amount of dust is varied in different working units, workers are stratified based on their working department and sample was proportionally allocated for each stratum (raw material production department, burner and clinker department, cement mill and packing department and cleaners). The study participants were selected by using simple random sampling technique.

### Study variables

#### Dependant variable

Chronic respiratory symptoms.

#### Independent variables

- Socio demographic factors: age, sex, education and marital status
- Behavioral factors: smoking, utilization, PPE
- Environmental factors: working department, length of working hours, work experience (service year), training on respiratory health, past dust exposure history, energy used at home
- Occupational history: dust exposure in work places before employing in the cement factory, chemical/gas exposure
- Chronic respiratory diseases (co-morbidities): chronic bronchitis, tuberculosis, (TB), heart disease, asthma and lung cancer.

### Operational definitions

- Chronic respiratory symptoms: The development of one or more of the symptom/s of chronic cough, chronic phlegm, chronic wheezing, chronic shortness of breath and chronic chest tightness which last/s at least three months in one year.
- Chronic Cough: Experience of cough as much as 4–6 times per day occurring for most days of the week (≥4 days) for at least three months in one year.
- Chronic Phlegm: It is sputum expectoration as much as twice a day for most days of the week (≥4 days) for at least three months in one year.
- Chronic Wheezing: A condition of causing a wheezy or whistling sound during inspiration/expiration at least three months in a year occasionally apart from that caused by a cold or acute upper respiratory infection.
- Chronic Chest tightness: In the past one year, chest pain that kept off work with phlegm.
- Chronic Shortness of breath: It is divided into 5 grades with the following definitions:
  - Grade 0: No breathlessness except with strenuous exercise;
  - Grade 1: Breathlessness when hurrying on the level ground or walking up a slight hill at least three months in a year.
  - Grade 2: Walking slower than people of the same age on the level because of breathlessness or need to stop for breath when walking at own pace or level at least three months in a year.
  - Grade 3: Stopping for breath after walking about a certain distance or a few minutes on the level ground at least three months in a year.
  - Grade 4: Too breathless to leave the house or breathless when dressing or undressing at least three months in a year.
- Smoking habit :
  - Never smokers: workers who used no cigarette.
  - Current smokers: workers who smoked at the time of the study or had stopped smoking less than one year before.
  - Ex-smokers: workers who had quit at least 1 year before the survey.
- Occupational (past dust exposure) history: any work experience on dusty environment before the current working position.
- Chronic respiratory disease: respiratory disease like TB, chronic bronchitis, lung cancer, and heart disease that could be developed before and identified by physicians.

### Data collection procedures

Data were collected through pre tested and structured questionnaires adopted form British Medical Research Council (BMRC) adult respiratory assessment questions [15]. Dust exposure, hygienic practices and use of personal protective equipments (PPE) were observed at the work site by using observational cheek list. The questionnaire contains issues about demographic factors, personal behavior, environmental factors, occupational history, chronic respiratory diseases, and respiratory symptoms, mainly cough, phlegm, shortness of breath, wheezing, and duration of respiratory symptoms.

### Data processing and analysis

The collected data were checked, coded and entered to epidemiological information package (EPi-info 3.5.1) and exported to statistical package for social sciences (SPSS) version 20 for further analysis. For most variables, data were presented as frequencies and percentages. Univariate logistic regression analysis was used primarily to check which independent variables are associated with the dependent variable individually. Variables found to have association (*p* < 0.2) with the dependent variables were then analyzed by multiple logistic regression for controlling the possible effect of confounders and finally the variables which had significant association were identified on the basis of AOR with 95 % CI and p< 0.05.

## Ethical considerations

The ethical issues of this study were confirmed by the ethical review committee of the University of Gondar and awarded permission letter. The copy of permission letter was submitted for the manager of the plant and data were collected after getting informed verbal consent from the manager and workers. Manager and workers were informed about the purpose of the study. Confidentiality was granted for the information collected from each study participants and privacy during interview was ensured.

## Results

### Socio-demographic characteristics of participants

Out of the total 404 respondents, 264(65.3 %) were males and 140(34.7) were females. The mean age was 28.69 with ± 8.07 standard deviation and ranging from 18 to 60 years. 211 (52.2 %) of participants were in the age group of 18–29, 106(26.2 %) of participants were attending grade eight and below and about 193(47.8 %) participants were married (Table 2).

**Table 2:** Socio - demographic information of Dejen cement factory workers, 2015

Variables		Frequency	Percent
Sex	Male	264	65.3
	Female	140	34.7
Age	18-29	211	52.2
	30-44	110	27.2
	45^+^	83	20.5
Educational status	≤ Grade 8	106	26.2
	Secondary education (9–12)	199	49.3
	Diploma and above	99	24.5
Marital status	Married	193	47.8
	Single	186	46.0
	Widowed and divorced	25	6.2

### Working environment and behavioral factors

363(89.9 %) out of the participants worked ≤ 48 h per week and 222(55 %) had work experience of five years or less. Only 126(31.4 %) employees used piece of cloths instead of respirator /dust mask as PPE and 77(19.1 %) had training about occupational health and safety. 47(11.6 %) participants were cigarette smokers and around 104 (25.7 %) used clean energy sources, of which 33(8.2 %) used electricity and 71(17.6 %) fuel gases at home (Table 3).

**Table 3:** Reported work environment and behavioral factors of participants in Dejen cement factory, March 2015

Variables		Number (n)	Percent (%)
Working Departments	Raw mill	170	42.1
	Burner and clinker	101	25.0
	Cement mill	133	32.9
Working hours per week	≤48	363	89.9
	>48	41	10.1
Service year	≤5	222	55.0
	>5	182	45.0
Use of PPE	yes	126	31.2
	No	278	68.8
Training on respiratory health	yes	77	19.1
	No	327	80.9
Smoking cigarette (current and ever smokers)	yes	47	11.6
	no	357	88.4
Energy used at home	Electricity and fuel gases	104	25.7
	Biomass	300	74.3

### Occupational history and previous chronic respiratory disease

**Fig. 1:**
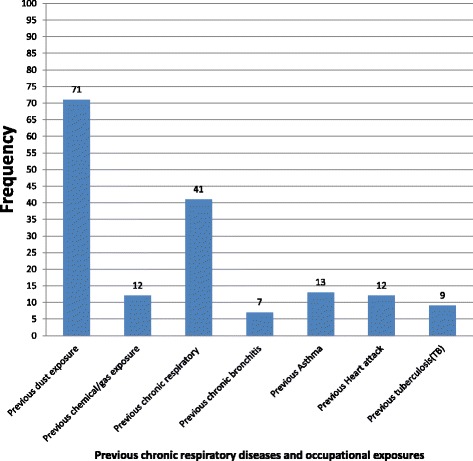
Previous chronic respiratory diseases and occupational exposures of participants in Dejen cement factory, March 2015

71(17.6 %) and 12(3 %) participants were exposed to dusty working conditions and chemicals/gas working environments, respectively, before they were employed to this cement factory. Few workers, 41(10.1 %), reported the presence of chronic respiratory disease identified by physicians before and after being employed in cement factory (Fig. 1).

### Work place observation

The survey indicated that cement dust was accumulated on the walls and floors of the working areas, and especially very high dust accumulated in the cement mill and packing department.

There were no employees observed using proper personal protective mask/respirator. The organization provided piece of cloth which was tied on the neck of the workers for protecting from the entry of dust particles to the oral and nasal cavity of the workers. Most of them did not use even those pieces of cloths “PPE” due to lack of supply. Some of them did not use the provided piece cloths since they believed that it reduces performance, was not comfortable, and inefficient for protecting the dust particles.

Most females used the provided piece of cloth in the work place. Even though the organization did not provide PPE, females also used their own cloths for protecting themselves from cement dust. Females were also compliant to safety and health rules and regulations in the organization while males were ignorant. Post work bathing, and post work change of clothes, were more practiced by females than male workers in Dejen cement factory.

In the plant, dust absorber/sucker had not been working for the last two years before data collection and the factory used open belt transport system from which dust particles easily dropped to the working area. This increased the dust accumulation in the working area.

### Chronic respiratory symptoms

**Fig. 2:**
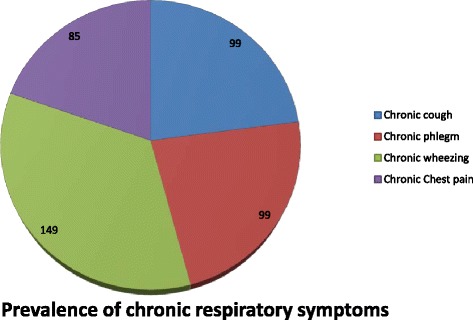
Prevalence of chronic respiratory symptoms among Dejen cement factory workers, March 2015

**Fig. 3:**
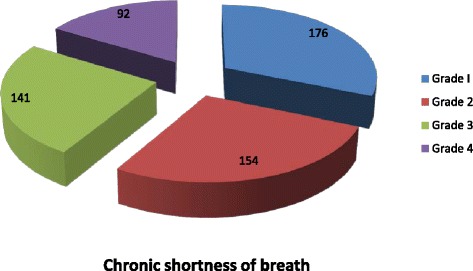
Shortness of breath among Dejen cement factory workers, March 2015

The prevalence of chronic respiratory symptoms among workers in Dejen cement factory was 62.9 % with prevalence of chronic cough 24.5 %,chronic wheezing 36.9 %, chronic phlegm 24.5 %, chronic chest pain 21.0 % and chronic shortness of breath 38.6 % (Figs. 2 and 3).

### Factors associated with chronic respiratory symptoms

Sex of participants, age, education level, working department, service years in the factory, training on respiratory health, smoking and previous chronic respiratory disease were significant both in univariate (p < 0.2) and multivariable(p < 0.05) analysis, while energy used at home was significant in univariate analysis, but insignificant in multivariable analysis. On the other hand, marital status, working hours per week, use of PPE, previous dust and chemical exposure were not associated with chronic respiratory symptoms in univariate analysis (*p* < 0.2) in Dejen cement factory.

Sex of participants was significantly associated with chronic respiratory symptoms among Dejen cement factory workers. Males were more likely to develop chronic respiratory symptoms (AOR = 2.07, 95 % CI = 1.18, 3.63) than females.

Workers aged ≥ 45 years were more likely to develop chronic respiratory symptoms (AOR = 4.20, 95 % CI = 1.94, 9.12) than workers in the age category 18–29 years old.

Workers whose education level was grade 8 or below were more likely to develop chronic respiratory symptoms than workers whose education level was diploma and above (AOR = 4.07, 95 % CI = 1.86, 8.92).

Working departments were significantly associated with chronic respiratory symptoms in the cement factory workers. Employees engaged in cement mill (AOR = 3.72, 95 % CI = 1.92, 7.21) were more likely to develop chronic respiratory symptoms than employees engaged in raw mill department.

Service year was also significantly associated with chronic respiratory symptoms. Workers who had work experience greater than five years had the odds of developing chronic respiratory symptoms 5.44 times more likely (AOR = 5.44, 95 % CI = 3.09,9.59) than workers with work experience less than or equal to five years in the cement factory.

Workers who had no training on occupational safety and health about respiratory problems related to dust had the odds of developing chronic respiratory symptoms 2.73 times more likely (AOR = 2.73,95 % CI = 1.41,5.29) than those workers who had got training.

Smokers developed chronic respiratory symptoms 5.38 times more likely (AOR = 5.38, 95 % CI = 1.42, 20.39) than non smokers.

Workers who had previous chronic respiratory diseases experienced chronic respiratory symptoms more likely than workers who were free from previous chronic diseases (AOR = 7.79, 95 % CI = 2.02, 30.04) (Table 4).

**Table 4:** Associated factors and chronic respiratory symptoms among cement factory workers in Dejen town, Amhara region, Ethiopia, 2015(n = 404)

Variable	Chronic respiratory symptoms		COR (95 % CI)	AOR (95 % CI)
	Yes	No		
Sex				
Male	187	77	2.65(1.73,.4.05)	2.07(1.18,3.63)*
Female	67	73	1.00	1.00
Age (years)				
14-29	99	112	1.00	1.00
30-44	86	24	4.05(2.39,6.87)	3.56(1.80,7.02)***
45^+^	69	14	5.58(2.96,10.52)	4.20(1.94,9.12)***
Educational status				
≤ Grade 8	77	29	3.46(1.93,6.20)	4.07(1.86,8.92)***
Secondary school (Grade 9–12)	134	65	2.69(1.64,4.41)	2.63(1.35,5.12)**
TVET and above	43	56	1.00	1.00
Working department				
Raw mill	76	94	1.00	1.00
Burner and clinker	68	33	2.55(1.52,4.26)	2.28(1.18,4.43)*
Cement mill	110	23	5.92(3.44,10.17)	3.72(1.92,7.21)***
Service year				
≤5	102	120	1.00	1.00
>5	152	30	5.96(3.72,9.56)	5.44(3.09,9.59)^***^
Training on respiratory health				
Yes	33	44	1.00	1.00
No	221	106	2.78(1.67,4.62)	2.73(1.41,5.29)**
Smoking status				
Never smokers	211	146	1.00	1.00
Ever Smokers	43	4	7.44(2.61,21.17)	5.38(1.42,20.39)**
Previous chronic respiratory disease				
Yes	38	3	8.62(2.61,28.45)	7.79(2.02,30.04)***
No	216	147	1.00	

Note: *: p ≤ 0.05, **: p ≤ 0.01, ***:p ≤ 0.001

## Discussion

This study revealed that sex, age, educational status, working department, work experience, training on occupational health and safety, cigarette smoking and previous chronic respiratory diseases were determinant factors for the development of chronic respiratory symptoms among Dejen cement factory workers.

This study revealed that the prevalence of chronic respiratory symptoms among Dejen cement factory workers was 254(62.9 %) with 95 % CI = 58.4, 68. The result of this study was more or less similar to the study conducted in cement factory workers in north Shewa, Ethiopia which was 66.2 % [16]. The result of this study was greater than that of the study done in India cement factory workers which was 54.4 % [6]. These differences might be due to effective control measures such as enclosed belt transport system in the work site where the previous study was conducted.

The prevalence of chronic cough was 24.5 %, chronic wheezing 36.9 %, chronic chest pain 21.0 %, chronic phlegm 24.5 %, and chronic shortness of breath 38.6 %. This result was lower than that of a study conducted in cement factories in Ethiopia that reported the prevalence of cough 73 %, phlegm 73.7 %, shortness of breath 71.1 % and chest pain 44.7 % [17]. This difference may be due to selection of participants of the previous study that were cleaners, who were more exposed groups. This may overestimate the prevalence of chronic respiratory symptoms. On the other hand, the result of this study was higher than that of the study conducted in cement factories in UAE and Taiwan, with prevalence of cough 19.5, 19.4 %, phlegm 14.8, 17.6 %, wheezing 2, 7.6 % and shortness of breath 4.7, 8.7 % respectively [18, 19]. These differences may be due to the safety concern and effective control measures such as modified filter and enclosed belt transport system for reduction of dust exposure of employees in these countries.

This study disclosed that males were more likely to develop chronic respiratory symptoms than females (AOR = 2.07, 95 % CI = 1.18, 3.63). This finding was in line with the studies conducted in Ethiopia and Zambia [16, 20]. This may due to the fact that females were in compliance with the control measures and instructions given by responsible bodies while males were reluctant as observed in work place survey. Thus, females were more prone to hygienic principles than males. Among females post work bathing, post work changing of cloths and washing of hands and face were more common. It was also observed that female workers were using their own piece of cloths for the prevention of their hairs, mouth and nose from cement dust more than male workers even though the organization failed to provide PPE.

We found that workers aged 45 and above years were more likely to develop chronic respiratory symptoms than workers aged 18–29 years (AOR = 4.20 95 % CI = 1.94, 9.12). This finding was consistent with a study conducted in Iran [21] but inconsistent with another one conducted in Ethiopia which reported that cleaners were younger but more likely to develop respiratory symptoms than manufacturing workers comparatively older than cleaners [17]. This discrepancy may due to the difference in the amount of dust exposure between the two comparative groups in the previous study and may also depend on the number of service years.

Educational status was another socio demographic factor which was statistically significant in this study. Workers who cannot write and read as well as primary school educated workers (grade 8 or below) were more likely at risk of developing chronic respiratory symptoms than workers who got a diploma or above levels of education (AOR = 4.07, 95 % CI = 1.86, 8.92). This result was in line with studies done in Zambia and UAE [20, 22]. Higher education provides the skills and knowledge about the means of protecting themselves from health effects associated with their work.

The working department was the main determinant factor for the development of chronic respiratory symptoms. This study found that workers in cement mill (AOR = 3.72, 95 % CI = 1.92, 7.21), burner and clinker (AOR = 2.28, 95 % CI = 1.18, 4.43) were more likely to develop chronic respiratory symptoms than workers in raw mill department. This statistical significance was due to the difference in dust concentration among different working departments (cement mill and raw mill) and the presence of smoke in the burner and clinker were observed during work place survey. It may also associate with the size of dust particles found in the working departments. The dust particles in raw mill were comparatively larger than those found in cement mill, burner and clinkers [23]. Therefore, workers in cement mill, burner and clinker departments were exposed to dust particles which were small enough to pass through nasal and oral cavities of employees. As a result, chronic respiratory symptoms were more frequent in these workers compared to those of raw mill department. The result of this study was consistent with the study conducted in Nigeria and Iran [14, 24] but inconsistent with findings of the study conducted in India [9]. This discrepancy may be due to reshuffling of workers in different shifts to different working departments in the previous study.

The study also revealed that a service length (work experience) >5 years was significantly associated with the development of chronic respiratory symptoms (AOR = 5.44, 95 % CI = 3.09, 9.59) deriving from increased dust accumulation associated with prolonged exposure. This result was consistent with the study done in India [6].

The present study also found that PPE use was not statistically significant in relation to the development of chronic respiratory symptoms (AOR = 1.42, 95 % CI = 0.92, 2.19). This finding was inconsistent with studies done in Tanzania and Nigeria [24, 25], and the difference may be due to the quality of PPE provided, that was piece of cloths instead of face mask or respirator, to the inconsistent use and cleaning of PPE (especially male workers) and to adequate supply of PPE in the present study as observed during work place observation.

Training on occupational health and safety related to dust was statistically significant in the present study. Cement factory workers who had no training were more likely to develop respiratory symptoms than workers who had got training (AOR = 2.73, 95 % CI = 1.41, 5.29). This finding was consistent with studies done in Egypt and Norway [11, 25]. The main reason for this result might be that training changes the attitude of workers towards respiratory health problems, and provides the skills and knowledge about the means of protecting themselves from health effects associated with dust work environments.

Ever smokers were more likely to develop chronic respiratory symptoms than workers who had never smoked (AOR = 5.38, 95 % CI = 1.42, 20.39). This result was consistent with the studies done in Iran, India and Italy [6, 21, 26], but it was inconsistent with another study done in Iran, which reported no difference in respiratory symptoms between smokers and non smokers [21]. This result might be due to the difference in the frequency and duration of smoking, and number of cigarettes smoked.

## Conclusions

This study found that chronic respiratory symptoms were more prevalent among cement factory workers in Dejen cement factory than in cement factories of developing Asian countries.

Sex, age, education level, long work experience in dusty working environments (service year), smoking habit, exposure to dust (work department) and previously identified chronic respiratory symptoms increased the odds of developing chronic respiratory symptoms, while higher education, effective dust control measures and training on occupational health and safety related to respiratory health problems were important determinant factors for maintaining the respiratory health of workers engaged in dusty working environments.
